# Neighborhood social reciprocity and mental health among older adults in China: the mediating effects of physical activity, social interaction, and volunteering

**DOI:** 10.1186/s12889-019-7385-x

**Published:** 2019-08-02

**Authors:** Ruoyu Wang, Hongsheng Chen, Ye Liu, Yi Lu, Yao Yao

**Affiliations:** 1 School of Geography and Planning, Sun Yat-Sen University, Xingang Xi Road, Guangzhou, 510275 China; 2 Guangdong Key Laboratory for Urbanization and Geo-Simulation, Sun Yat-Sen University, Xingang Xi Road, Guangzhou, 510275 China; 3 School of Architecture, Southeast University, Si-Pai-Lou Road No. 2, Nanjing, 210096 China; 4 Department of Architecture and Civil Engineering, City University of Hong Kong, Hong Kong, SAR China; 5 City University of Hong Kong Shenzhen Research Institute, Shenzhen, 518057 China; 6 School of Geography and Information Engineering, China University of Geosciences, Wuhan, 430074 China

**Keywords:** Neighborhood social reciprocity, Physical activity, Social interaction, Volunteering, Mental health

## Abstract

**Background:**

This study aims to investigate the mechanisms through which neighborhood social reciprocity influences older adults’ mental health in China.

**Methods:**

This study used data from the 2011–2015 waves of the China Health and Retirement Longitudinal Study. It estimated the effects of neighborhood social reciprocity on older adults’ mental health and tested the mediating effects of the frequencies of physical activity, social interaction with neighbors, and volunteering experience.

**Results:**

The results indicated that more neighborhood social reciprocity related to better mental health. The effects of the three mediators were statistically significant and enhanced mental health. In addition, the effects of the mediators were strengthened by neighborhood social reciprocity, and vice versa.

**Conclusions:**

In China, neighborhood social reciprocity influenced older adults’ mental health directly and through the mechanisms of the frequencies of physical activity, social interaction with neighbors, and volunteering experience.

## Background

Recent studies have suggested that depression has become one of the top three diseases that cause disability [1], and this has been attracting significant attention, particularly in developing countries [2–4, 54]. Depression has been found to lead to various physical diseases, such as cardiovascular diseases [5, 6] or stroke [7], and it even increases mortality rates [8, 9]. Older people tend to be more likely than younger people to suffer from depression, and thus, more attention should be paid to this age group [10–12]. There are age-related reasons for this difference in mental health. First, older people tend to be less involved than younger people in the labor market, which might weaken their social ties, which in turn might cause a sense of loneliness and worsen their mental health [12]. Second, older people might suffer from functional limitations that prevent frequent physical activity, which might negatively influence their mental health [12, 13].

Neighborhood social reciprocity is an important aspect of neighborhood social capital, and many previous studies have found that it benefits people’s mental health status [2–4, 14–20]. Neighborhood social reciprocity might influence mental health through three main mechanisms [15]. First, it has been found to encourage people to adopt healthy behaviors, such as physical activities [15, 21]. Second, studies have found that it increases the diffusion of health-related information through increased social interaction [15, 22]. Third, neighborhood social reciprocity likely exerts informal control over individuals’ compliance with norms relevant to mental health, such as encouraging residents to participate in voluntary or charity work [15, 22–24].

### The mechanisms through which neighborhood social reciprocity influences mental health

Neighborhood social reciprocity might encourage people to adopt healthy behaviors, such as various physical activities, that benefit their mental health [15, 21, 25]. However, for many older people, physical capacities degrade with age, and they are relatively likely to fear that injuries will result from physical activity, and therefore, they avoid those activities [13]. However, older people who live in neighborhoods with high levels of neighborhood social reciprocity often believe that they can obtain the support and assistance of their neighbors in times of need, which might encourage them to participate in physical activities. Neighborhood social reciprocity also tends to encourage residents to work together to maintain public facilities and spaces in their neighborhoods. Consequently, older people in those communities might have access to higher quality sports facilities and more open spaces for physical activity than older adults in neighborhoods with low levels of reciprocity [15, 21, 25, 26].

Second, neighborhood social reciprocity might enhance the diffusion of health-related information by encouraging social interaction among neighbors [15, 18, 27]. Many older people have weaker social ties than younger people after they leave the workforce [12, 28], but neighborhood social reciprocity might overcome that loss through increased interpersonal interactions and mutual helping in the neighborhood. In the context of neighborhoods with high social reciprocity, residents are more likely to obtain and learn about health-related information from their neighbors compared to residents living in neighborhoods with low social reciprocity [15, 18, 27]. Neighbors might offer emotional support, comfort, and instrumental support, such as financial resources, all of which benefit mental health [15, 18, 27]. Hypothesis 2 was developed based on this reasoning.

Third, as mentioned earlier, neighborhood social reciprocity likely exerts informal control over individuals’ compliance with norms relevant to mental health, such as encouraging residents to take part in voluntary or charity work [15, 22–24]. Residents living in neighborhoods with high social reciprocity are more likely to realize their role as contributing citizens in the process of self-identification, because they get more support in such neighborhoods, which in turn encourages them to help others and understand their value to others [23, 24]. Older adults’ physical status may restrain their participation in voluntary work [12], but neighborhoods with high social reciprocity are better organized, which can help older adults get more involved in voluntary work [24]. Volunteering experience may improve older adults’ mental well-being since it provides them with a feeling of belonging, a sense of connection with others, and helps them realize their own identity [29]. Moreover, volunteering experience can help reduce people’s sense of loneliness and social isolation, so it can be particularly protective for widowed or retired older adults [30].

### The contextual effect of neighborhood social reciprocity on the relationship between healthy behaviors and mental health among older adults

Because neighborhood social reciprocity might encourage the diffusion of health-related information, it might condition the effects of healthy behaviors on mental health [15, 17, 18, 27, 31]. Healthy behaviors, such as participating in physical activities and interpersonal interactions, might be more beneficial for mental health in neighborhoods with high as opposed to low neighborhood social reciprocity. If neighborhood social reciprocity increases residents’ health-related knowledge (through enhanced diffusion of health-related information), it is reasonable to conclude that they would be more likely to know how to maximize the positive effects of adopting healthy behaviors, such as physical activities, in neighborhoods with high levels of social reciprocity. For example, De Silva et al. [31] systematically reviewed studies on the relationship between social support and mental illness and found that residents in neighborhoods with high neighborhood social capital were more likely than their lower social capital counterparts to engage in the physical activities most likely to support their health. Fisher et al. [25] found that residents engaged in physical activity in neighborhoods with high neighborhood social capital were more likely than those in neighborhoods with low social capital to be correctly advised on exercise techniques, such as workout movements. Therefore, we hypothesized that neighborhood social reciprocity indirectly influences mental health.

**Fig. 1:**
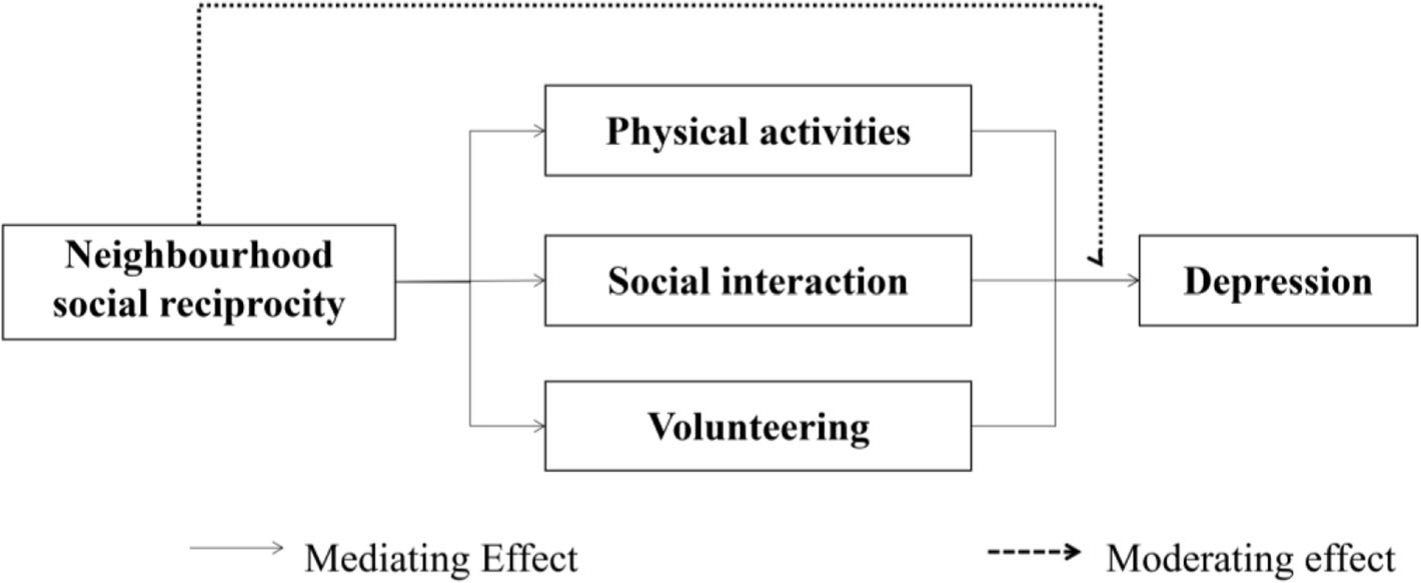
The theoretical framework

This study investigated three mechanisms that link neighborhood social reciprocity to older adults’ mental health (specifically, self-reported depressive symptoms) using data derived from the 2011, 2013, and 2015 China Health and Retirement Longitudinal Study (CHARLS), a nationally representative study on older adults’ health issues in China. It focused on the mediating roles of the frequencies of physical activity, social interaction with neighbors, and the odds of having volunteering experience in the relationship between neighborhood social reciprocity and mental health. It further considered the extent to which neighborhood social reciprocity moderated those mediating effects. The theoretical framework of these relationships is shown in Fig. 1.

This study contributes to the previous literature in four ways. First, it systematically analyzed the effects of neighborhood social reciprocity on mental health using longitudinal data. Second, it examined the mechanisms through which neighborhood social reciprocity positively influenced older adults’ mental health. Third, it tested the conditional effects of neighborhood social reciprocity on the relationships of the frequencies of physical activity, social interaction with neighbors, and volunteering experience with mental health. Last, this study used a large and heterogeneous sample of nationally representative longitudinal data on older adults in China.

## Methods

### Study sample

This study’s data were derived from the 2011, 2013, and 2015 waves of the CHARLS. The National Development Research Institute of Peking University, which conducts the CHARLS, employed the probability-proportional-to-size sampling technique. First, 150 city-level divisions were randomly chosen from 30 provinces. Then, 450 neighborhoods were randomly chosen from the 150 city-level divisions. About 40 people older than 45 years were randomly drawn from each neighborhood. Because this study focused on older adults, we analyzed respondents older than 60 years and dropped cases with invalid or missing data, resulting in a final sample size of 24,620 person-year records (mixed longitudinal data).

### Data

#### Dependent variable

The CES-D 10 (10-item Center for Epidemiologic Studies Depression Scale, [32]) is a proven measure of mental health that is valid and reliable for older adults in many countries [33]. This study’s dependent variable (mental health) was measured using the CES-D 10. The measure is a 10-item Likert-type scale questionnaire that asks respondents to assess their mental states, such as their happiness, hopelessness, and so on, during the past week. The response categories range from 0 = *rarely or none of the time* to 3 = *most or all of the time*, and the item on respondents’ positive feelings is reverse coded. The summed CES-D 10 items’ responses indicate the severity of mental health, and higher scores mean more severe mental health problems (range: 0–30). The Cronbach’s alpha of the CES-D 10 in this study was 0.95.

### Independent variables

#### Neighborhood social reciprocity

Respondents were asked to indicate how often during the past 12 months they had helped their neighbors who do not live with them and who did not pay them for that help (*almost daily*; *almost weekly*; *not regularly*; *never*). Following previous studies [3, 4, 15, 17, 34, 35], the responses were categorized into a dichotomous indicator contrasting those with high social reciprocity (almost daily or almost weekly) to those with low social reciprocity (not regularly or never). Neighborhood social reciprocity was indicated as the proportion (ratio) of persons in the respondent’s neighborhood that reported high social reciprocity.

#### Mediators

Three mediators were tested in this study: frequencies of physical activity, social interaction with neighbors, and participating in voluntary work. The frequency of physical activity was measured by self-reported weekly physical exercise time (in hours). To obtain a normal distribution, the log form of the variable was computed and analyzed. Following previous studies [36], social interaction with neighbors was measured by responses to a question about the frequency of interaction with neighbors in respondents’ neighborhood. The response options were on a four-point scale where 1 = *never*, 2 = *not regularly*, 3 = *almost weekly*, and 4 = *almost daily*. Last, volunteering experience was measured by responses to a question about whether respondents have done voluntary or charity work (1 = *yes*, 0 = *no*).

### Covariates

The effects of some socioeconomic and demographic factors were controlled for in the analysis, including gender, age, educational attainment, marital status, household size, annual household income, rural/urban residence, cigarette use, alcohol use, self-reported physical health status, functional ability, and medical insurance. Because we focused on older adults, the influence of the extent of respondents’ functional abilities was controlled for. Functional ability was indicated as *limited* or *not limited* based on the Activities of Daily Living (ADL). Respondents’ ADL scores were derived from responses to 13 questions on daily activities, such as dressing, bathing, and so on. Respondents that reported a problem with at least one of the listed activities were categorized as *limited* [37]. Table 1 presents the distributions of all the variables used in the analysis.

**Table 1:** Descriptive statistics, CHARLS 2011–2015 (*n* = 24,620)

Variables	Proportion/Mean (SD)
Dependent variable	
CES-D score (range: 0–30)	8.31 (6.32)
Independent variables	
Neighbourhood social reciprocity ratio (0–1.0)	0.12 (0.10)
Social reciprocity (%)	
High	11.36
Low	88.64
Physical activity (weekly exercise time in hours)	9.41 (17.15)
Frequency of social interaction with neighbours (range: 1–4)	1.78 (1.17)
Volunteering (%)	
Yes	3.02
No	96.98
Control variables	
Gender (%)	
Male	48.92
Female	51.08
Age (in years)	67.25 (6.29)
Educational attainment (%)	
Primary school or less	81.93
High school	16.52
College or more	1.55
Marital status (%)	
Single, divorced or widowed	16.95
Married and living with spouse	79.45
Married and living apart from spouse	3.60
Household size (number of persons)	3.08 (2.05)
Annual household incomes per capita (CNY)	
Residence (%)	
Urban neighbourhood	40.81
Rural neighbourhood	59.19
Cigarette use (%)	
Current smoker	36.15
Current non-smoker	63.85
Alcohol use (%)	
Yes	33.58
No	66.42
Physical health status (%)	
Reported health problem	65.34
No problems	34.66
ADL limited (%)	
Yes	52.25
No	47.75
Medical insurance (%)	
Yes	26.74
No	73.26

### Statistical analyses

A three-level multilevel model was used to test the hypotheses and analyze the relationship between neighborhood social reciprocity and mental health [38] because of the hierarchical structure of the longitudinal data. The following model was estimated:

$$ {\displaystyle \begin{array}{c}\mathrm{CES}-{\mathrm{D}}_{\mathrm{ti}\mathrm{j}}={\upbeta}_0+{\upbeta}_1\mathrm{Neighbourhood}\ {\mathrm{social}\ \mathrm{reciprocity}}_{\mathrm{j}}+{\upbeta}_2{\mathrm{Physical}\ \mathrm{activities}}_{\mathrm{ti}\mathrm{j}}\\ {}+{\upbeta}_3{\mathrm{Social}\ \mathrm{interaction}}_{\mathrm{ti}\mathrm{j}}+{\upbeta}_4{\mathrm{Volunteering}}_{\mathrm{ti}\mathrm{j}}+{\upbeta}_5{\mathrm{Covariates}}_{\mathrm{ti}\mathrm{j}}\\ {}+{\upbeta}_6{\mathrm{Covariates}}_{\mathrm{ti}}+{\upvarepsilon}_{\mathrm{ti}\mathrm{j}}+{\upmu}_{\mathrm{ij}}+{\upvarphi}_{\mathrm{j}}\end{array}} $$

where *t* represents time (wave), *i* represents individuals, and *j* represents neighborhoods. β_0_ is the intercept. *Neighborhood social reciprocity*_j_ represents a vector of neighborhood-level variables of neighborhood social reciprocity. *Physical activities*_tij_, *Social interaction*_tij_ and *Volunteering*_tij_ are mediators. *Covariates*_tij_ represents a vector of time-variant covariates. *Covariates*_ti_ represents a vector of time-invariant covariates, and ε_tij_, μ_ij_, and φ_j_ represent random errors within individuals, between individuals, and between neighborhoods, respectively.

The variance inflation factor (VIF, < 3) was used to ensure that multicollinearity did not bias the results. First, Model 1 estimated the bivariate effect of neighborhood social reciprocity on CES-D scores. Second, Models 2 to 4 estimated the effects of neighborhood social reciprocity on the three mediators (physical activity, social interaction, and volunteering experience) (Hypothesis 1–3). Third, to further test Hypotheses 1 to 3, we estimated the influence of neighborhood social reciprocity on CES-D scores and added each mediator separately (physical activity, social interaction, and volunteering experience) (Models 5 to 7). Fourth, Model 8 estimated the effect of neighborhood social reciprocity on CES-D scores and all three mediators (physical activity, social interaction, and volunteering experience) to test the multiple mediation effect [39] of the three mediators (to verify Hypotheses 1–3). Last, cross-level interaction terms were added to Model 8 for Model 9 to estimate the contextual effects of neighborhood social reciprocity on the relationships between the three mediators and CES-D scores (Hypothesis 4). Sensitivity analysis was conducted, such as by redefining high social reciprocity as those who reported “almost daily through almost weekly” or “not regularly,” excluding respondents aged above 85 (oldest-old) and respondents who were ill abed, but the results were not substantively altered (results available on request). Statistical analyses were carried out in STATA 15.1.

## Results

### The effect of neighborhood social reciprocity on mental health

Table 2 shows that the effect of neighborhood social reciprocity on CES-D scores was negative (β = − 0.175, SE = 0.084), meaning that respondents with high neighborhood social reciprocity had low CES-D scores (β = − 0.598, SE = 0.288), which indicates that they reported better mental health. Males had lower CES-D scores than females (β = − 1.481, SE = 0.099); CES-D scores decreased with age (β = − 0.049, SE = 0.007); and respondents with higher educational attainment had lower CES-D scores (high school β = − 0.750, SE = 0.111; college or more β = − 0.969, SE = 0.324). Married respondents and those not living with their spouse had lower CES-D scores (β = − 1.046, SE = 0.112), and CES-D scores negatively related to household size (β = − 0.065, SE = 0.018). The (log) household income per capita negatively related to CES-D scores (β = − 0.035, SE = 0.011), and urban residents had lower CES-D scores than rural residents (β = − 1.666, SE = 0.170). Smokers, less healthy respondents, and respondents with at least one functional limitation had higher CES-D scores than their counterparts (β = 0.372, SE = 0.091; β = 1.278, SE = 0.084; and β = 2.787, SE = 0.080, respectively). Respondents who had medical insurance had higher CES-D scores than those who did not (β = 0.518, SE = 0.093).

**Table 2:** The effect of neighbourhood social reciprocity on mental health; three-level multilevel longitudinal correlation regression analysis of neighbourhood social reciprocity, individual characteristics and CES-D score (*n* = 24,620 in 450 neighbourhoods)

Variable	Model 1	
	Beta	(SE)
Fixed effects		
High social reciprocity (ref: low social reciprocity)	−0.175**	(0.084)
Neighbourhood social reciprocity	−0.598**	(0.288)
Male (ref: female)	−1.481***	(0.099)
Age	−0.049***	(0.007)
Educational attainment (ref: primary school or less)		
High school	−0.750***	(0.111)
College or more	−0.969***	(0.324)
Marital status (ref: single, divorced or widowed)		
Married and living with spouse	−0.326	(0.228)
Married and living apart from spouse	−1.046***	(0.112)
Household size	−0.065***	(0.018)
Logarithm of household income per capita	−0.035***	(0.011)
Urban neighbourhood (ref: rural neighbourhood)	−1.666***	(0.170)
Cigarette use (ref: no)	0.372***	(0.091)
Alcohol use (ref: no)	−0.127	(0.088)
Physical health status (ref: no problems)	1.278***	(0.084)
Functional ability (ref: not limited)	2.787***	(0.080)
Medical insurance (ref: no)	0.518***	(0.093)
Constant	12.062***	(0.525)
Random effects		
Var (Neighbourhoods)	2.394**	
Var (Individuals)	13.851**	
Var (Within individuals)	17.833**	
Number of years	3	
Akaike information criterion (AIC)	154,503.300	

*SE* standard error

* = *p* < .10, ** = *p* < .05, *** = *p* < .01

### The effect of neighborhood social reciprocity on the frequencies of physical activity, social interaction with neighbors, and volunteering

Table 3 shows the results of Model 2, which tested Hypothesis 2 and estimated the effects of neighborhood social reciprocity on the three mediators. Neighborhood social reciprocity positively influenced the frequency of physical activity (β = 0.293, SE = 0.131), and respondents with high social reciprocity reported more frequent physical activity (β = 0.192, SE = 0.031). There were positive relationships between neighborhood social reciprocity and the frequency of social interaction with neighbors (β = 0.024, SE = 0.011), and respondents with high social reciprocity reported more social interaction than respondents with low social reciprocity (β = 0.572, SE = 0.023). Model 3 (Table 3) indicates that neighborhood social reciprocity also positively related to respondents’ odds of having volunteering experience (Odds = 13.883, 95% CI = 4.456–43.257). Therefore, respondents with high social reciprocity are also more likely to have volunteering experience (Odds = 5.121, 95% CI = 4.055–6.464).

**Table 3:** The effects of neighbourhood social reciprocity on frequency of physical activity (Model 2), frequency of social interaction with neighbours (Model 3) and the odds of having volunteering experience (Model 3); three-level multilevel longitudinal models (*n* = 24,620 in 450 neighbourhoods)

Variable	Model 2		Model 3		Model 4	
	(log) Physical activity		Frequency of social interaction with neighbours		The odds of having volunteering experience	
	Beta	(SE)	Beta	(SE)	Odds	(95% CI)
High social reciprocity (ref: low social reciprocity)	0.192***	0.031	0.572***	0.023	5.121	4.055–6.464
Neighbourhood social reciprocity	0.293**	0.131	0.024**	0.011	13.883***	4.456–43.257
Akaike information criterion (AIC)	89,359.490		75,422.350		5831.849	

Models were fully adjusted. SE = standard error. *OR* odds ratio, *CI* confidence interval. * = *p* < .10, ** = *p* < .05, *** = *p* < .01

### The mediating effects of the frequencies of physical activity, social interaction with neighbors, and volunteering on the relationship between neighborhood social reciprocity and mental health

Table 4 presents the results of Models 5 to 9 on the analysis of the mediating effects of the frequencies of physical activity, social interaction with neighbors, and volunteering on the relationship between neighborhood social reciprocity and CES-D scores. Model 5 extended Model 1 to estimate the mediating role of the frequency of physical activity by including the measure of (log) physical activity. The result was that (log) physical activity negatively and significantly related to mental health (β = − 0.035, SE = 0.008), and the results of the Sobel test [40] confirmed that the frequency of physical activity significantly mediated the influence of neighborhood social reciprocity on CES-D scores (*z* = − 1.991, *p* = 0.046). Model 6 replaced (log) physical activity with the measure of the frequency of social interaction with neighbors, which also negatively related to CES-D scores (β = − 0.234, SE = 0.031), and the Sobel test result indicated that the frequency of social interaction with neighbors significantly mediated the relationship between neighborhood social reciprocity and mental health (*z* = − 2.096, *p* = 0.036). The results of Model 7 indicate that having volunteering experience was negatively related to CES-D scores, and a mediating effect was confirmed via the Sobel test (β = − 0.736, SE = 0.209, *z* = − 2.783, *p* = 0.005). Model 8 simultaneously estimated the mediating roles of the three mediators, which only slightly changed the results found in Models 5 to 7. The multiple mediation test [39] found that the three mediators collectively influenced the relationship between neighborhood social reciprocity and CES-D scores (*z* = − 3.232, *p* = 0.001). In sum, the proportion mediated by physical activity was 3.51%, social interaction was 2.84%, and volunteering was 6.02%. In the multiple mediation models, the mediators combined proportion accounted for 9.68%. In Model 9, the conditional effects of neighborhood social reciprocity were estimated by including multiplicative interaction terms between the frequencies of physical activity and social interaction with neighbors, and neighborhood social reciprocity. Table 4 shows that the effects of neighborhood social reciprocity depended on the frequencies of physical activity and social interaction with neighbors, and the coefficients of both were negative and statistically significant. In other words, neighborhood social reciprocity strengthened the effects of the frequencies of physical activity and social interaction with neighbors on CES-D scores toward better mental health. However, there was no evidence to support that neighborhood social reciprocity also moderated the relationship between volunteering and CES-D scores.

**Table 4:** The mediating effects of frequency of physical activity (Model 5), frequency of social interaction with neighbours (Model 6) and volunteering experience (Model 7) on the relationship between neighbourhood social reciprocity and CES-D score; the multiple mediation effect (Model 8); the conditional effects of neighbourhood social reciprocity depending on the effects of the mediators; three-level multilevel longitudinal models (*n* = 24,620 in 450 neighbourhoods)

	Model 5		Model 6		Model 7		Model 8		Model 9	
Mediation variable	(log) Physical activity		Frequency of social interactions with neighbours		The odds of having volunteering experience		Multiple mediation		Conditional mediation	
	Beta	(SE)	Beta	(SE)	Beta	(SE)	Beta	(SE)	Beta	(SE)
(Log) physical activity	−0.035***	0.008					− 0.032***	0.008	− 0.034***	0.009
Frequency of social interaction (neighbours)			−0.234***	0.031			−0.227***	0.031	−0.226***	0.031
Volunteering (ref: no)					−0.736***	0.209	−0.663***	0.209	−0.707***	0.228
High social reciprocity (ref: low social reciprocity)	−0.169**	0.084	−0.047**	0.022	−0.127**	0.064	−0.101**	0.050	−0.104**	0.050
Neighbourhood social reciprocity	−0.577**	0.282	−0.581**	0.287	−0.562**	0.280	−0.540**	0.269	−0.569**	0.272
Cross-level interaction terms										
Neighbourhood social reciprocity × (log) physical activities									−0.432**	0.211
Neighbourhood social reciprocity × frequency of social interactions with neighbours									−0.211**	0.098
Neighbourhood social reciprocity × Volunteering (ref: no)									−1.047	2.049
Akaike information criterion (AIC)	154,453.000		154,450.100		154,471.600		154,421.100		154,423.200	

Models were fully adjusted. *SE* standard error. * = *p* < .10, ** = *p* < .05, *** = *p* < .01

## Discussion

This study estimated the mediating effects of the frequencies of physical activity, social interaction with neighbors, and volunteering experience on the relationship between neighborhood social reciprocity and mental health among older adults in China. The results found that neighborhood social reciprocity influenced mental health toward better health, and all three mediators individually and collectively mediated and strengthened that relationship. In addition, the beneficial effects of the frequencies of physical activity and social interaction with neighbors were moderated by neighborhood social reciprocity.

In this nationally representative sample, neighborhood social reciprocity increased the frequencies of physical activity, social interaction with neighbors, and volunteering, which in turn related to lower CES-D scores (i.e., better mental health). The results support the findings of previous studies. First, neighborhood social reciprocity positively influenced physical activity, which might lessen mental health problems. Currently, square dancing is the most popular physical activity for older adults in China, and, because people who live in neighborhoods with high social reciprocity are probably more likely to maintain public spaces, they might have relatively more public space for square dancing activities as well [41, 42]. In addition, when people are confident of their neighbors’ reciprocity, they are less concerned about injuries during physical activities than are those who live in other neighborhoods, and this might increase participation [41–44].

This study also found that neighborhood social reciprocity positively related to the frequency of social interaction with neighbors, which influenced self-reported mental health. This finding probably relates to the fact that more neighborhood social reciprocity is characterized by more neighbors helping neighbors, which strengthens social ties and cohesion in the neighborhood [15, 22, 45]. China’s older adults often obtain health-related information from their neighbors, which is probably more frequent in neighborhoods with high social reciprocity, because sharing information is an important way in which people reciprocate [46]. Emotional support is probably more commonly offered and accepted as well, particularly for older adults who feel lonely [47, 48]. These behaviors help to improve mental health.

Neighborhood social reciprocity increased the odds of respondents having volunteering experience, which related to fewer mental health problems (lower CES-D scores). As an explanation of this finding, it might be possible that neighborhood social reciprocity exerts some social control on residents regarding giving back to the neighborhood, since they may receive much support in such a neighborhood. Previous studies in China have indicated that neighborhood social reciprocity may improve older adults’ volunteerism, because older adults are more likely to enjoy the benefits of volunteering in neighborhoods with high social reciprocity and they may get involved in voluntary services to contribute to their neighborhood in return [49–51]. Existing research has found that volunteering may also benefit older adults’ mental health in China [41, 52] Due to retirement, older adults in China may not recognize their own value and may become lonely, but they may derive perceived rewards and satisfaction with the experience of volunteering [41, 52]. Based on empirical evidence in China, this study confirms that volunteering mediates the relationship between neighborhood social reciprocity and depression.

The cross-level interaction effects between neighborhood social reciprocity and the mediators on CES-D scores found conditional effects of neighborhood social reciprocity, in which the influences of the mediators to the benefit of mental health were strengthened by neighborhood social reciprocity. Acquisition of health-related information might be easier or faster because these social interactions are relatively intimate, which might encourage sharing useful health-related information [15]. Moreover, the beneficial effect of physical activity was strengthened by neighborhood social reciprocity, which could also be explained by the increase in useful health-related information, because with more health-related information, older adults are more likely to know what physical activities to engage in and how to maximize the benefits of physical activity for their mental health.

This results of this study have several policy implications. First, it is clear that efforts to improve neighborhood social reciprocity would benefit older adults’ mental health. Second, policy makers are advised to create open public spaces in neighborhoods to encourage older adults to participate in group-based physical activities [26, 53], such as square dancing. Third, lectures, workshops, and so on for older adults on health-related topics might increase their knowledge and encourage them to increase their social interaction with neighbors and volunteering experience.

This study has several strengths. First, it used data collected from 450 neighborhoods across 150 city-level divisions in China, which are large in scale and have heterogeneous environmental settings. Second, this study used longitudinal data in three waves, thereby ensuring the robustness of the results. Third, the present study focused on both moderating and mediating effects, so the mechanisms through which neighborhood social reciprocity influences mental health among older adults can be further understood.

Despite the current study’s contributions, it also has some limitations. First, the three mediators in the analysis do not represent all the possible influences on CES-D scores or neighborhood social reciprocity, and other important mechanisms should be analyzed. Second, the data cover three longitudinal waves over a short period, which might not be long enough to convincingly support causal interpretations of the results regarding neighborhood social reciprocity and mental health for older adults in China. Third, the CHARLS only collects older adult respondents’ information, so it only indicates neighborhood social reciprocity specifically among older adults, instead of the general neighborhood social reciprocity for the whole neighborhood. Last, due to the relatively high mortality rate for older adults, the missing data in this study may cause some potential bias.

## Conclusion

This study found clear evidence that neighborhood social reciprocity negatively influences CES-D scores, meaning that neighborhood social reciprocity is good for older adults’ mental health. The relationship was mediated by the frequencies of physical activity, social interaction with neighbors, and volunteering experience. Neighborhood social reciprocity strengthened the beneficial effects of the mediators on mental health, and vice versa. We recommend that researchers not limit future studies to direct effects. In conclusion, neighborhood social reciprocity in China is important to older adults’ mental health through the mechanisms of physical activity, social interaction with neighbors, and volunteering experience.

## Data Availability

The datasets used in this study are publically available at http://charls.pku.edu.cn/zh-CN.

## Abbreviations

AIC: Akaike information criterion
CES-D: Center for Epidemiologic Studies Depression Scale
CHARLS: China Health and Retirement Longitudinal Study
VIF: Variance inflation factors
